# Genome Sequences of the Delta Variant (B.1.617.2) and the Kappa Variant (B.1.617.1) Detected in Morocco

**DOI:** 10.1128/MRA.00727-21

**Published:** 2021-09-30

**Authors:** Marouane Melloul, Taha Chouati, Mouhssine Hemlali, Sanaa Alaoui Amine, Nadia Touil, Hicham Elannaz, Khalid Ennibi, Mohammed Youbi, Mouad Merabet, Abdelkrim Meziane Bellefquih, Jalal Nourlil, Abderrahmane Maaroufi, Elmostafa El Fahime

**Affiliations:** a Molecular Biology and Functional Genomics Platform, National Center for Scientific and Technical Research (CNRST), Rabat, Morocco; b Genomic Center for Human Pathologies (GENOPATH), Faculty of Medicine and Pharmacy, Mohammed V University, Rabat, Morocco; c Research and Biosafety Laboratory, Mohammed V Military Teaching Hospital, Rabat, Morocco; d DELM (Epidemiology and Disease Control Department), Ministry of Health, Rabat, Morocco; e Laboratory of Virology, Pasteur Institute, Casablanca, Morocco; f Pasteur Institute, Casablanca, Morocco; DOE Joint Genome Institute

## Abstract

Here, we report the identification and coding-complete genome sequences of severe acute respiratory syndrome coronavirus 2 (SARS-CoV-2) strains obtained from patients with COVID-19. The strains identified belong to variant of concern B.1.617.2 and variant of interest B.1.617.1.

## ANNOUNCEMENT

Human severe acute respiratory syndrome coronavirus 2 (SARS-CoV-2), a member of the family *Coronaviridae* and genus *Betacoronavirus*, was first identified in the city of Wuhan, Hubei Province, China, in late December 2019 (1). SARS-CoV-2 was first reported in Morocco in March 2020, and since then, an exponential rise in the number of cases has been experienced by the country, resulting in 549,844 cases and 9,418 deaths as of 16 July 2021 (http://www.covidmaroc.ma). We announce here the coding-complete genome sequences of SARS-CoV-2 strains recovered from two patients who tested positive using a reverse transcription-quantitative PCR assay (RT-qPCR). The study was approved by the Biomedical Research Ethics Committee of Mohammed V University, Faculty of Medicine and Pharmacy, Rabat, Morocco (approval number 17/20, delivered on 12 June 2020).

During surveillance, nasopharyngeal swab samples were obtained on 22 April 2021 from an asymptomatic patient who had traveled from India to Morocco (sample I) and from an asymptomatic Moroccan patient living in Casablanca (sample II). The viral nucleic acids were extracted using the MagPurix viral RNA extraction kit according to the manufacturer’s recommendations (Zinexts Life Science, China). The patients were confirmed as being infected with SARS-CoV-2 by RT-qPCR using the GeneFinder COVID-19 Plus RealAmp kit with threshold cycle (*C_T_*) values of 10 (N gene), 12 (E gene), and 14 (RdRp) for sample I and 12, 14, and 15 for the N, E, and RdRp genes, respectively, for sample II.

Whole-genome sequencing of the specimens was performed using Sanger sequencing technology (Applied Biosystems, USA) at the National Center for Scientific and Technical Research (Rabat). Following the protocol for the SuperScript VILO cDNA synthesis kit (Invitrogen, Thermo Fisher Scientific, USA), cDNAs were obtained and amplified using PCR with 38 specific primer pairs, covering the coding-complete genome according to the CDC protocol (2). The primers used cover the SARS-CoV-2 genome from positions 64 to 29857, with 37 overlapping regions between the PCR products. Following the primer walking strategy, the PCR amplification yielded 38 amplicons, which were sequenced using a BigDye Terminator cycle sequencing kit v3.1 (Applied Biosystems) and purified using the BigDye XTerminator purification kit, according to the manufacturer’s instructions (Applied Biosystems). Consensus sequences were generated by assembling the obtained sequences with the Wuhan-Hu-1 reference sequence (GenBank accession number NC_045512.2) using Unipro UGENE v38.1 software (3). All tools were run with default parameters.

Coding-complete genome sequences of 29,715 bp and 29,818 bp were obtained for samples I and II, respectively, with GC contents of 37.95% and 37.99%. Using the Pangolin Web application (https://pangolin.cog-uk.io/), the strains under study were assigned to lineages B.1.617.2 and B.1.617.1 for samples I and II, respectively (4); these lineages were first reported in India in October 2020. Sequence analysis using the CoV-GLUE Web application (http://cov-glue.cvr.gla.ac.uk/) (5) revealed that the spike protein of variant of concern (VOC) B.1.617.2 contains eight missense changes (T19R, G142D, R158G, L452R, T478K, D614G, P681R, D950N) with an in-frame deletion of two amino acids at positions 156 and 157 (E156del and F157del), while variant of interest (VOI) B.1.617.1 contains eight missense changes (T95I, G142D, E154K, L452R, E484Q, D614G, P681R, Q1071H) (Table 1); some of these changes are of great concern. Both variants have the L452R mutation located in the receptor-binding motif (RBM) within the receptor-binding domain (RBD). This amino acid change was reported to strengthen the SARS-CoV-2 infectivity and decrease its sensitivity to neutralizing antibodies (6, 7). The two variants also have a mutation at amino acid 681, substituting a proline (P) for an arginine (R). Position 681 is located near the furin cleavage site between subunits S1 and S2, which is important for the pathogenesis of the virus (8).

**TABLE 1:** Nonsynonymous mutation profiles of the Delta variant, lineage B.1.617.2 (strain I), and the Kappa variant, lineage B.1.617.1 (strain II)^*a*^

Gene^*b*^	Strain	Genomic changes	Amino acid changes
ORF1ab	I	C1191T, C5051T, C5184T, C6539T, C9891T, T11418C, G14122A, **C14408T**, G15451A, C16466T, C20320T	P309L, P1596S, P1640L, H2092Y, A3209V, V3718A, G4620S, **P4715L**, G5063S, P5401L, H6686Y
	II	C4965T, A11201G, **C14408T**, G16852T, G17523T, A20396G, T20401G	T1567I, T3646A, **P4715L**, G5530C, M5753I, K6711R, S6713A
S	I	C21618G, **G21987A**, A22028G, 22029del–22034del, **T22917G**, C22995A, **A23403G**, **C23604G**, G24410A	T19R, **G142D**, R158G, E156del–F157del, **L452R**, T478K, **D614G**, **P681R**, D950N
	II	C21846T, **G21987A**, G22022A, T22917G, G23012C, **A23403G**, **C23604G**, A24775T	T95I, **G142D**, E154K, **L452R**, E484Q, **D614G**, **P681R**, Q1071H
ORF3a	I	**C25469T**	**S26L**
	II	**C25469T**	**S26L**
M	I	T26767C	I82T
	II	T26767G	I82S
ORF7a	I	C27739T, C27752T, **T27638C**	L116F, T120I, **V82A**
	II	**T27638C**	**V82A**
ORF8	I	C27972T, 28248del–28253del	Q27,^*c*^ D119del–F120del
N	I	**G28881T, G29402T**, A28461G, G29427A	**R203M, D377Y**, D63G, R385K, S26L
	II	**G28881T, G29402T**	**R203M, D377Y**

a Del, deletion. Bold text indicates mutations that appear in both variants.

b ORF, open reading frame; S, spike; M, membrane; N, nucleocapsid.

c Stop codon.

SARS-CoV-2 variants continue to emerge, spreading worldwide with devastating consequences. Large-scale genomic surveillance is crucial to further investigate the spread of the virus and identify variants that were recently introduced in Morocco.

### Data availability.

The SARS-CoV-2 genome sequences were submitted to the GISAID database under the identifiers EPI_ISL_2110643 and EPI_ISL_2966236 and to NCBI GenBank under the accession numbers MZ208926 and MZ571142. The raw Sanger reads of the two strains are available on the Zenodo platform under the record number 5471509.
